# Cytogenetics and DNA barcode reveal an undescribed *Apareiodon* species (Characiformes: Parodontidae)

**DOI:** 10.1590/1678-4685-GMB-2018-0066

**Published:** 2019-06-27

**Authors:** Emanoel Oliveira dos Santos, Geize Aparecida Deon, Rafael Bonfim de Almeida, Ezequiel Aguiar de Oliveira, Viviane Nogaroto, Hugmar Pains da Silva, Carla Simone Pavanelli, Marta Margarete Cestari, Luiz Antonio Carlos Bertollo, Orlando Moreira-Filho, Marcelo Ricardo Vicari

**Affiliations:** 1 Departamento de Genética, Programa de Pós-Graduação em Genética, Universidade Federal do Paraná, Curitiba, PR, Brazil; 2 Departamento de Biologia Estrutural, Molecular e Genética, Universidade Estadual de Ponta Grossa, Ponta Grossa, PR, Brazil; 3 Departamento de Genética e Evolução, Universidade Federal de São Carlos, São Carlos, São Paulo, SP, Brazil; 4 Laboratório de Citogenética e Genética Animal, Instituto de Biociências, Universidade Federal de Mato Grosso, Cuiabá, MT, Brazil; 5 Núcleo de Pesquisas em Limnologia, Ictiologia e Aquicultura (Nupélia), Universidade Estadual de Maringá, Maringá, PR, Brazil

**Keywords:** Chromosomal differentiation, hidden diversity, repetitive DNA, sex chromosomes

## Abstract

Parodontidae is a small group of fish and some species are particularly difficult to identify due to the lack of sufficiently consistent morphological traits. Cytogenetically, the species possess 2n = 54 chromosomes and are either sex-homomorphic or sex-heteromorphic (regarding its chromosomes). We evaluated data on color, tooth morphology, cytogenetics, and mitochondrial markers (COI) in *Apareiodon* specimens from the Aripuanã River (Amazon basin) and the results were compared to other congeneric taxa. Morphological results show an overlap of body color and tooth morphology to other known *Apareiodon*. The cytogenetics data showed that the 2n = 54 chromosomes, 50 m/sm + 4 st and, a ZZ/ZW sex chromosome system in *Apareiodon* sp. are common to other species of the genus. However, the number and chromosomal localization of the 45S ribosomal and p*Ph*2004 satellite DNA sites, in addition to W chromosome localization of the p*Ph*2004 appear to be exclusive cytogenetic features in *Apareiodon* sp. Our phylogenetic tree revealed well-supported clades and confirmed, by barcode species delimitation analysis, a new Molecular Operational Taxonomic Unit (MOTU) for *Apareiodon* sp. (Aripuanã River). As a whole, the above features support the occurrence of a new species of the *Apareiodon*, thus far unknown for the Parodontidae.

## Introduction

The family Parodontidae is currently composed of three genera: *Apareiodon* Eigenmann, 1916; *Parodon* Valenciennes, 1849 and *Saccodon* Kner, 1863 (Pavanelli, 2003), with 32 valid species (Eschmeyer and Fong, 2017). Its species can be differentiated from the other Characiformes by the following combined features: an edentulous lower jaw in the anterior region and spatulate mandible, pedunculated and multicuspided premaxillary teeth with wide distal border distributed in a single series, and by the absence of upper lip and fontanelle (Pavanelli, 2003). The genera are mainly characterized by variation in the number of undivided rays in the pectoral fins. However, the color pattern with one regular black longitudinal stripe or several vertical bands, in addition to the shape and number of the tooth cusps adjacent to the premaxillary symphysial tooth, can also be used in the species identification (Pavanelli, 2003).

Cytogenetic analyzes in Parodontidae revealed a conserved diploid number of 54 chromosomes, most of them meta/submetacentric, with few or none subtelocentric chromosome (Moreira-Filho *et al.*, 1980; Jesus and Moreira-Filho, 2000; Rosa *et al.*, 2006; Vicari *et al.*, 2006). Acrocentric chromosomes are an exception, only found in *A. affinis* of the lower Paraná River system (Jorge and Moreira-Filho, 2000, 2004; do Nascimento *et al.*, 2018). Differences in karyotypes, such as distinct karyotypic formulas, number and localization of the satellite DNA p*Ph*2004 and 18S and 5S rDNA sites, distribution of the heterochromatic bands and occurrence of sex chromosomes (Moreira-Filho *et al.*, 1984, 1985; Bellafronte *et al.*, 2011, 2012; Schemberger *et al.*, 2011, 2014, 2016; Ziemniczak *et al.*, 2014) appear as exclusive derived species features.

However, one of the most outstanding cytogenetic characteristics in the differentiation of Parodontidae species is the occurrence of sex chromosome systems. This way, besides species without heteromorphic sex chromosomes and with proto-sex ones, heteromorphic ZZ/ZW sex chromosomes and multiple sex systems of the ZZ/ZW_1_W_2_ type also occur (Schemberger *et al.*, 2011). Heterocromatization is commonly associated to the morphological differentiation of the W chromosome (Centofante *et al.*, 2002; Vicente *et al.*, 2003; Vicari *et al.*, 2006; Schemberger *et al.*, 2011; Bellafronte *et al.*, 2012). Accumulation of satellite sequences and transposable elements (TEs) were events responsible for molecular differentiation and erosion of the W chromosome gene activity, allowing the identification of pseudo-autosomal (PAR) and W specific (WSR) regions in Parodontidae (Schemberger *et al.*, 2014; Ziemniczak *et al.*, 2014). In addition, repetitive sequences also promoted the differentiation among autosomes, acting on the karyotypic diversification of this group (Bellafronte *et al.*, 2011; Schemberger *et al.*, 2011, 2014, 2016; Ziemniczak *et al.*, 2014; Traldi *et al.*, 2016; do Nascimento *et al.*, 2018).

Integrative cytogenetic and DNA barcoding studies were effective in detecting chromosome evolution and species richness within supposedly homogeneous taxa of *A. affinis* (do Nascimento *et al.*, 2018). Indeed, molecular analysis contributes to reveal a hidden biodiversity (Cunha *et al.*, 2017; Ramirez *et al.*, 2017; do Nascimento *et al.*, 2018) and has been widely used for identification and delimitation of neotropical fish species (Carvalho *et al.*, 2011; Pereira *et al.*, 2011, 2013; Ramirez and Galetti Jr, 2015; Machado *et al.*, 2016). In this sense, one of the most used genes for species identification is the mitochondrial cytochrome c oxidase subunit 1 (COI) gene, which was proposed by Hebert *et al.* (2003) as a DNA barcode methodology. Thus, DNA barcoding and population genetics can be used to define discrete genetic lineages, characterizing Molecular Operational Taxonomic Units (MOTUs) and/or revealing reciprocal monophyly (Floyd *et al.*, 2002).

In this study, morphological patterns, chromosomal markers, and DNA barcode analysis were used with the objective of identifying a probable undescribed *Apareiodon* species from the Aripuanã River, Mato Grosso State, Brazil, here named as *Apareiodon* sp. In addition, we discuss the chromosomal evolution and species diversification within the Parodontidae.

## Material and Methods

### Biological samples

Chromosome studies were performed on 32 specimens (23 males and 9 females) of *Apareiodon* sp., collected in the Aripuanã River, Amazon basin (10º09’57.8"S and 59º26’54.9" W). The chromosomal and DNA material was obtained from the Laboratory of Fish Cytogenetics of the Universidade Federal de São Carlos. The procedures were in agreement with the Ethics Committee for Animal Use of the Universidade Estadual de Ponta Grossa (Protocol: 29/2016). The fish were fixed in 10% formalin and, after 48 hours, preserved in 70% ethanol. The specimens were identified based mainly on a black band on the third distal edge of the dorsal fin, but also on the combination of other morphological features, such as a black lateral band without up and downwards projections, two maxillary teeth, premaxillary teeth with a rounded cutting edge and 9 to 11 cusps, among others. Voucher specimens were deposited at the Coleção Ictiológica of Núcleo de Pesquisas em Limnologia, Ictiologia e Aquicultura (Nupélia) of Universidade Estadual de Maringá (NUP 19988).

### Analysis of body color, symphysial teeth, and tooth cusps

Body color analysis was performed according to the method described by Pavanelli and Bristki (2003). Photos of unfixed and fixed specimens were used in order to ascertain the presence of the black dorsal-fin band and yellowish tone in *Apareiodon* sp. (Aripuanã River). Also, an extraction and analysis of the symphysial teeth was performed following do Nascimento *et al.* (2018). Teeth were photographed using a bright-field microscope (Olympus BX43) coupled to a DP72 CCD camera (Olympus) at 40x magnification for further counting of the cusps number.

### Chromosomal preparations

Metaphase chromosomes were obtained from kidney cells, after *in vivo* treatment with colchicine and conventional air-drying preparation (Bertollo *et al.*, 2015), and the chromosomal preparations in slides were submitted to conventional Giemsa staining 5% in phosphate buffer (pH = 6.8). Constitutive heterochromatin was detected by the C-banding method (Sumner, 1972). The images were captured with a microscope (Olympus BX43) coupled to a DP72 CCD camera (Olympus), edited, and arranged into karyotypes using Adobe Photoshop software CC 2015. Homologous chromosomes were paired and arranged into metacentric-submetacentric (m/sm) and subtelocentric (st) groups, according to Levan *et al.* (1964). Two arms were considered for each one of such chromosome types to determine the fundamental number (FN).

### Fluorescence *in situ* hybridization (FISH)

FISH was performed following Pinkel *et al.* (1986), and five types of probes were used to localize complementary sequences in the metaphase chromosomes of *Apareiodon* sp. The following two sequences were isolated by PCR from the total genome of *Apareiodon* sp., according to the respective authors: 5S rDNA (Barros *et al.*, 2017), 18S rDNA (Gross *et al.*, 2010). In addition, the *Parodon hilarii* satellite DNA probe named p*Ph* 2004 (Vicente *et al.*, 2003), the heterochromatic fraction of the W chromosome of *Apareiodon* sp., named W*Ap* (Vicari *et al.*, 2010), and a (GATA)n microsatellite probe (Traldi *et al.*, 2013) were used.

The 18S rDNA and (GATA)n probes were labeled using digoxigenin-11-dUTP hapten (Jena Bioscience), while 5S rDNA, p*Ph* 2004, and W*Ap* were labeled using biotin-16 -dUTP hapten (Roche Applied Science). A general FISH protocol was followed under a stringency condition of ~80% (2.5 ng/μL probe, 50% formamide, 2xSSC, 10% dextran sulfate, 42 °C for 16 h). Post-hybridization washes were done at high stringency (50% formamide at 42 °C for 20 min, 0.1xSSC at 60 °C for 15 min, and 4xSSC 0.05% Tween at room temperature for 10 min). Streptavidin Alexa Fluor 488 (Molecular Probes) and an anti-digoxigenin rhodamine fab fragment (Roche Applied Science) were used for probe detection. The chromosomes were stained with DAPI (0.2 μg/mL) present in Vectashield mounting medium (Vector) and analyzed under epifluorescence microscopy.

### Molecular analysis

For genomic DNA extraction, liver samples were used following the CTAB (cetyltrimethylammonium bromide) method of MURRAY AND Thompson (1980). DNA samples were used to amplify the barcode region of the mitochondrial gene *cytochrome C oxidase subunit I* (COI) by PCR. Amplification of the COI sequence was performed using the primers *Fish F1* and *Fish R1* (Ward *et al.*, 2005). The reaction mix was composed by 1x *Taq* Reaction buffer (200 mM Tris pH 8.4, 500 mM KCl), 40 μM dNTPs, 2 mM MgCl_2_, 2 U of *Taq* DNA polymerase (Invitrogen, Carlsbad, USA), 0.2 μM of each primer, and 100 ng of DNA template. The following reaction program was used: initial denaturation for 2 min at 94 °C, 35 cycles of 94 °C for 1 min, 52 °C for 40 s, 72 °C for 1 min, and a final extension at 72 °C for 10 min. After amplification, the samples were purified and submitted to nucleotide sequencing using an ABI-Prism 3500 Genetic Analyzer (Applied Biosystems)*.*

COI sequences obtained for *Apareiodon* sp. from the Aripuanã River and for *A. vittatus* (Jangada River) were deposited in GeneBank (access numbers: MG827218 - MG827229). Additionally, COI sequences of *A. piracicabae* (Passa Cinco River), *A. affinis* (Cuiabá River), and *A. affinis* (Passa Cinco River) described by Bellafronte *et al.* (2013) were used. All sequences were first analyzed with Geneious 8.1.9 software (Kearse *et al.*, 2012) and aligned using ClustalW algorithm, where possible sequencing errors were checked using the BLAST tool against the data deposited in GenBank (NCBI).

Estimates of genetic distances were obtained using the MEGA software 7.0.14 (Kumar *et al.*, 2016) under the Kimura-2-Parameters (K2P) evolution model (Kimura, 1980). Gene flow, haplotype (h), and nucleotide (π) diversity analyses were performed by DnaSP software (Librado and Rozaz, 2009). Population structuring and analysis of molecular variance (AMOVA) (Excoffier *et al.*, 1992), were performed by the Arlequin 3.5.2.2 software (Excoffier and Lischer, 2010). The PopArt 1.7 program was used to generate a haplotype network through the median joining criterion (Leigh and Bryant, 2015). Structural analysis was performed by assignments of each individual to the respective populations using Bayesian inference in Structure 2.3.4 software (Pritchard *et al.*, 2000) and Bayesian Analysis of Population Structure - BAPS 6 (Corander *et al.*, 2004; Corander and Marttinen, 2006). The Bayesian inference tree was generated with the MrBayes 3.2 program (Huelsenbeck and Ronquist, 2001; Ronquist and Huelsenbeck, 2003).

## Results

### Color and teeth

**Figure 1:**
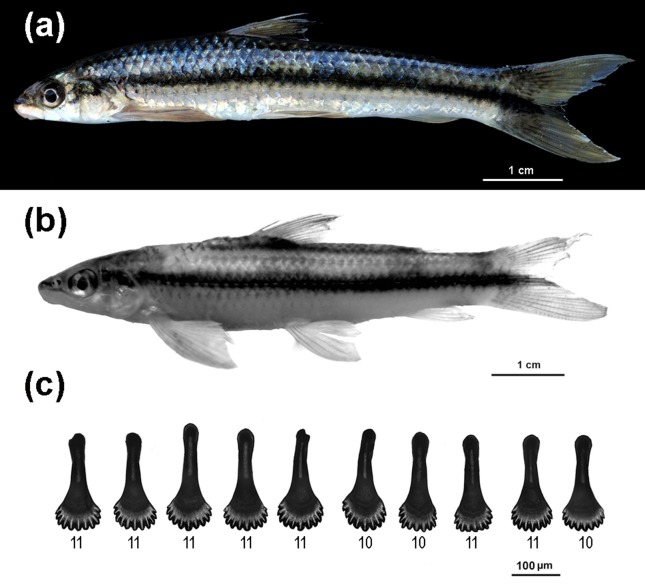
Photograph of the *Apareiodon* sp. from the Aripuanã River: In (a) coloured image showing a regular black longitudinal stripe extending from head to tail; the fins have a yellowish tone (pectoral, dorsal, caudal) and the end of the black stripe; In (b) black and white image showing details of the fins. In (c) symphesial teeth images showing the tooth morphology and cusps number. The number in each tooth is the number of cusps.

The *Apareiodon* sp. individuals showed a regular black longitudinal stripe extending from head to tail. The fin had a yellowish tone (pectoral, dorsal, caudal) and the end of the black stripe (Figure 1a). The symphysial teeth showed rounded edges, bearing 10 to 11 cusps (Figure 1b).

### Cytogenetics

**Figure 2:**
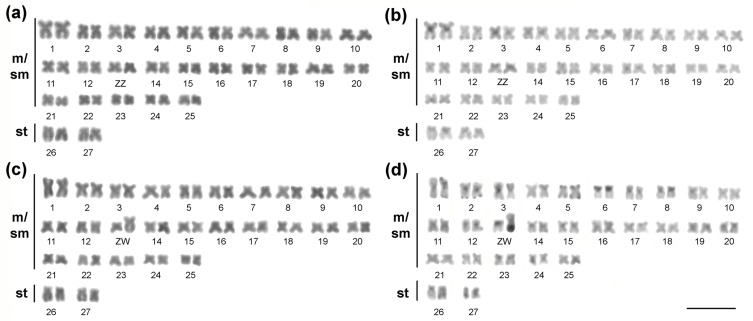
Karyotypes of the males (a, b) and females (c, d) *Apareiodon* sp. specimens. Giemsa staining (a, c) and C-banding (b, d). Bar = 10 μm.

All specimens had 2n=54 chromosomes, FN=108, with 50 m/sm + 4st for both sexes. However, the 13^th^ pair appeared heteromorphic in females, revealing a ZZ/ZW sex chromosome system (Figure 2). Both Z and W chromosomes were metacentric in form, but the W one was much larger, corresponding to the second largest chromosome of the complement (Figure 2). The heterochromatin bands were mainly pericentromeric, in addition to some terminal bands (Figure 2b, d). While the Z chromosome presented only a small proximal heterochromatic band in the short arm, the W chromosome had most of its long arm heterochromatic (Figure 2b, d).

**Figure 3:**
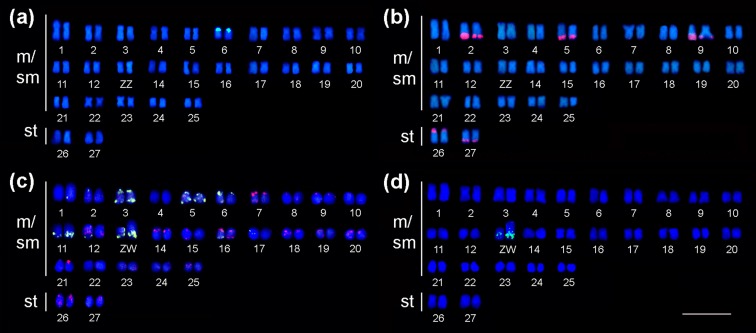
Karyotypes of the *Apareiodon* sp. submitted to probing for *in situ* localization: in (a) 5S rDNA (green); (b) 18S rDNA (red); (c) p*Ph* 2004 (green) and (GATA)n (red) and; (d) W*Ap* probe (green). Bar = 10 μm.

FISH analysis with the 5S rDNA probe showed that this multigene family is localized in the p arm of pair 6 (Figure 3a). The *in situ* localization of 18S rDNA evidenced ten terminal clusters in pairs 2, 5, 9, 26 and 27, all in the long arms with exception of the pair 26 (Figure 3b). The DNA p*Ph*2004 satellite was clustered in the terminal region of pairs 3, 5, 6 and, 11, in addition to ZW sex chromosomes, as well as the GATAn sequence although not in the same chromosome pairs neither in the Z and W chromosomes (Figure 3c). In addition, the heterochromatic W*Ap* probe was also localized in the proximal region of the q arm of the Z chromosome and in a great extent of the q arm of the W chromosome (Figure 3d).

### Molecular analysis

Twenty-six fragments of the COI gene from different *Apareiodon* individuals were analyzed. The sequences did not show insertions, deletions, stop codons, or sequencing errors. The sequences contained 706 nucleotides, with 183 variable sites, and a nucleotide diversity of *π* = 0.13475. The haplotype number considered was 16, with a haplotype diversity of h = 0.93231.

**Figure 4:**
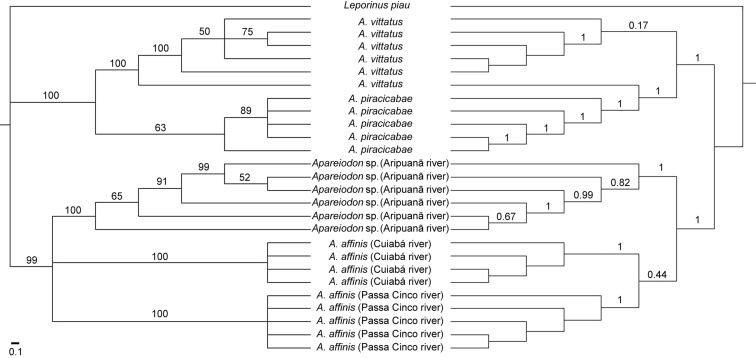
Species tree showing phylogenetic relationships from the five analyzed species/populations of Parodontidae. To the left, topology for Maximum Likelihood (numbers on the branches are bootstrap values). To the right, Bayesian tree (numbers on the branches are posterior probability). The scale bar indicates nucleotide substitutions per site.

Genetic K2P divergence among species ranged from 5.9 to 23.4% and, when considering only *Apareiodon* sp. (Aripuanã River) in comparison to the other *Apareiodon* species, the K2P genetic distance was 20.6 to 23.4% (Table 1). The maximum likelihood tree and Bayesian analysis considering the substitution TIM2+G model (Posada, 2003) given by Jmodeltest revealed five main consistent branches: *Apareiodon* sp. (Aripuanã River), *A. vittatus*, *A. piracicabae*, *A. affinis* (Cuiabá River), *A. affinis* (Passa Cinco River) (Figure 4). *Leporinus piau* was used as out-group in the tree and demonstrated the ancestral relationship with all species analyzed in-group.

**Table 1:** Estimates of evolutionary divergence over sequence pairs among groups.

Species	*Apareiodon* sp. (Aripuanã)	*A. vittatus*	*A. affinis* (Cuiabá)	*A. affinis* (Passa Cinco)	*A. piracicabae*
*Apareiodon* sp. (Aripuanã)					
*Apareiodon vittatus*	0.217 (± 0.02)				
*Apareiodon affinis* (Cuiabá)	0.215 (± 0.02)	0.228 (± 0.02)			
*Apareiodon affinis* (Passa Cinco)	0.205 (± 0.02)	0.206 (± 0.02)	0.059 (± 0.01)		
*Apareiodon piracicabae*	0.233 (± 0.02)	0.151 (± 0.01)	0.098 (± 0.01)	0.077 (± 0.01)	
Intraspecific divergence	0.042 (± 0.01)	0.033 (± 0.00)	0.00 (± 0.00)	0.000 (± 0.00)	0.000 (± 0.00)

The number of base substitutions per site from averaging overall sequence pairs between groups are shown. Kimura-2-Parameters genetic distance mean and standard error. Analyses were conducted using the Kimura 2-parameter model. The analysis involved 26 nucleotide sequences. Codon positions included were 1st+2nd+3rd. All positions containing gaps and missing data were eliminated. There was a total of 441 positions in the final dataset.

**Figure 5:**
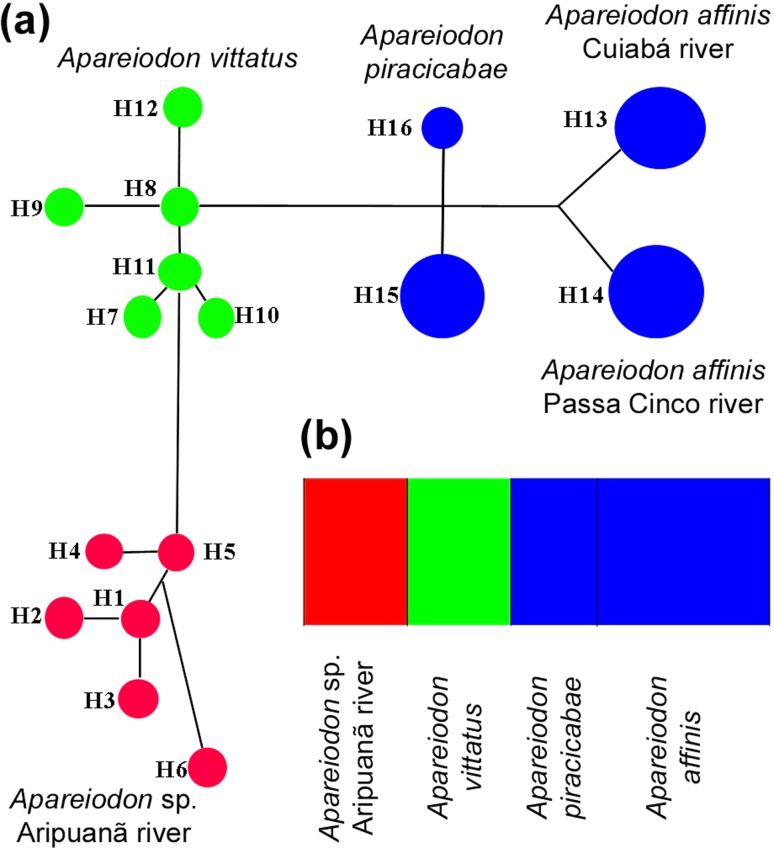
Molecular data of *Apareiodon* sp. from the Aripuanã River. In (a), the Median Joining haplotype network of *Apareiodon* sp., *A. vitattus*, *A. piracicabae*, and *A. affinis* using COI gene data. The haplotypes are shown in different colors according to species or MOTU. In (b), BAPs data for the five analyzed species/populations of Parodontidae. The MOTU for *Apareiodon* sp. (Aripuanã River) is shown in red color.

The barcode sequences of the *Apareiodon* species analyzed were also used for the haplotype network construction. This network showed that there is no haplotype overlap among the species, and that the haplotypes of same species are more closely related to each other when compared with others (Figure 5a). AMOVA analysis returned values of Φ_ST_ = 0.69829. Regarding populational structuration inference, the analysis generated by BAPS indicated that there is no sequence overlap, thus evidencing independent groups (Figure 5b).

## Discussion

Parodontids are mostly rheophilic, and therefore absent in the lower regions of the Amazon basin. However, the *Apareiodon* species from the upland rivers of the Amazon basin are morphologically less similar to *Apareiodon* sp. from the Aripuanã River than to congeners from the La Plata basin. In the body, the wide longitudinal stripe of *Apareiodon* sp. showed relatively similar patterns to *Apareiodon* species from the Paraná-Paraguai system (*A. vittatus*, *A*. *piracicabae*, and *A. affinis*), except for the black stripe at the dorsal fin. Ingenito and Buckup (2005) inferred that these similarities in color pattern between several Parodontidae species are suggestive of close relationship, but should be evaluated in a phylogenetic context. In addition to body color, the symphysial teeth with rounded edges and 9 to 11 cusps overlap in shape and cusp numbers with those of *A. piracicabae* from the upper Paraná and upper São Francisco river basins (Pavanelli, 2006).

Nevertheless, cytogenetic and DNA barcode data show a clear speciation scenario. Although having a diploid number (2n = 54), a chromosomal formula (50 m/sm + 4 st), a FN value (108), and localization of 5S rDNA sequences in a single chromosomal pair, similar to other species of the genus (Bellafronte *et al.*, 2011; Schemberger *et al.*, 2011; Traldi *et al.*, 2016), a set of chromosomal markers were able to differentiate *Apareiodon* sp. from other congeneric species. Regarding chromosomal differences in *Apareiodon* sp., this species shows an exclusive number of five chromosome pairs bearing major rDNA sequences (18S rDNA probe) among the Parodontidae species; the W chromosome differs from the other Parodontidae with a ZZ/ZW sex chromosome system by the exclusive localization of the satellite p*Ph*2004 in the q arm, and by having a high number of p*Ph*2004 sites in exclusive chromosomal localization of the karyotype.

The 18S rDNA sites occupies the long arm terminal region of a single large subtelocentric chromosome pair in most species of *Apareiodon* (Moreira-Filho *et al.*, 1984, 1985; Rosa *et al.*, 2006; Vicari *et al.*, 2006). Even though there are some few exceptions for multiple 18S rDNA sites occuring in *Apareiodon* (Bellafronte *et al.*, 2009; Traldi *et al.*, 2016), there is no case with such a high number as in *Apareiodon* sp., which may be related to some distinct chromosomal rearrangements, such as inversions, translocations and transposon-mediated transpositions (Symonová *et al.*, 2013; Barbosa *et al.*, 2017). On the other hand, the 5S rDNA localization is a conserved trait in the Parodontidae, where the chromosome pair bearing these sequences appears as homologue in most species (Bellafronte *et al.*, 2009, 2011). Additional 5S rDNA sites are a rare occurrence, and only *Parodon nasus* and *A. affinis* showed this site at a different chromosome and position (Bellafronte *et al.*, 2009; Traldi *et al.*, 2016; do Nascimento *et al.*, 2018).

It was proposed that the differentiation of the ZZ/ZW sex system in Parodontidae is related to a paracentric inversion, in which the terminal W*Ap* repetitive sequence was transposed to the proximal region of the short arm of a metacentric pair, with subsequent amplification leading to the differentiation of the W chromosome (Centofante *et al.*, 2002; Vicente *et al.*, 2003; Schemberger *et al.*, 2011). Apparently, only *Apareiodon hasemani* presents a distinct stage of heterochromatinization on the p arm (Bellafronte *et al.*, 2012). In *Parodon moreirai* and *P. hilarii,* the Z chromosome presents satellite DNA at the terminal region of the q arm, while the W chromosome has p*Ph*2004 in the PRA (pseudo-autosomal region located at p arm), and W*Ap* and GATAn sequences at the WSR (W specific region located at q arm). The W chromosome of *Apareiodon* sp. differs by the exclusive localization of the satellite p*Ph*2004 in the q arm. However, the WSR of *Apareiodon* sp. keeps its repetitive sequences concerning the W*Ap* probe identical to other Paradontidae, with exception of the GATAn expansion, which does not show accumulation in this chromosome.

In addition to the exclusive condition of the p*Ph*2004 site in the q arm in the W chromosome of *Apareiodon* sp., this species possesses a high number of p*Ph*2004 sites (14) distributed in three autosomal and ZW chromosomes. Karyotypes presenting numerous p*Ph*2004 sites were described for *P. nasus*, *P. hilarii*, and *A. affinis* (Centofante *et al.*, 2002; Vicente *et al.*, 2003; do Nascimento *et al.*, 2018), but the chromosomal sites localization is quite divergent in relation to *Apareiodon* sp. (Aripuanã River). All these cytogenetic features corroborate with the barcode, haplotypic network, and BAPs data, which demonstrate differentiation and absence of gene flow between *Apareiodon* sp. and the other *Apareiodon* species with a similar body color pattern.

DNA barcode studies have been useful in demonstrating genetic divergence among Parodontidae species, such as *A. affinis*, *A. ibitiensis*, *A. piracicabae*, *A. vitattus*, *A. vladii*, *Apareiodon* sp. from Verde River, *P. nasus* and *P. moreirai* (Bellafronte *et al.*, 2013), all well morphologically supported clades. The comparative population studies of *A*. *affinis* were equally informative, evidencing that the species from the upper Paraná differ from those of the lower Paraná River basin, and that three populations from the lower Paraná system (Uruguai, Paraguai, and Cuiabá rivers) display divergent chromosomal features, as well as low values of genetic divergence, which could indicate possible parapatric speciation processes in progress (do Nascimento *et al.*, 2018). In our study, *Apareiodon* sp. represents the only Amazonian species (Madeira basin) that was compared to other *Apareiodon* species with a similar color pattern (*A. piracicabae*, *A. vittatus*, *A. affinis*), all of them from the Paraná River basin, and the results showed that *Apareiodon* sp. from the Aripuanã river belong to an undescribed species in the literature.

According to Lundberg (1998), the modern separation between the Paraná and Amazon systems occurred about 30 Myr ago. Since then, headwater captures among rivers from these hydrographic systems were also documented to have occurred during the last 10 Myr (Lundberg, 1998). The results of K2P genetic divergence demonstrated that *Apareiodon* sp. is distant by 20-23% from other *Apareiodon* evaluated, these high K2P values being compatible with distinct fish species (Hebert *et al.*, 2003; Ward *et al.*, 2005, 2009). Thus, although probable fauna interchanges occurred between the Paraná and Amazon systems, the population structure analysis, in addition to K2P genetic divergence evidenced that *Apareiodon* sp. appears as a distinct biological entity inside Parodontidae.

The haplotype network shows no overlap between *Apareiodon* sp., *A. vittatus*, *A. piracicabae*, and *A. affinis* COI haplotypes, corroborating absence of gene flow and isolation among these species. A similar case was demonstrated for *Leporinus friderici* as a new MOTU in the Madeira basin, which also demonstrated gene flow isolation to other *Leporinus friderici* in the Amazon and Paraná basins (Silva-Santos *et al.*, 2018). Within the Amazon basin itself, *Apareiodon* sp. seems to be morphologically different from congeners, and apparently it is not found outside the Madeira River basin (Pedroza *et al.*, 2012; Fernandes *et al.*, 2013).

Our phylogenetic analyses showed that specimens, although morphologically similar, compose particular groups in distinct branches with high support values, indicating clades that have ancient segregations. The close relationship of *Apareiodon* sp. and *A. affinis*, from the Paraná-Paraguai system, indicates some current or recent connections between the basins, corroborating data by Brea and Zucol (2011) and Dagosta and De Pinna (2017). In this scenario, population genetics data, in addition to particular cytogenetic characteristics, indicate that *Apareiodon* sp. from the Aripuanã River differentiated from morphologically close congeneric species and appears as a new Molecular Operational Taxonomic Unit (MOTU) within the Parodontidae.
